# Comparison of dental plaque reduction after use of electric toothbrushes with and without QLF-D-applied plaque visualization: a 1-week randomized controlled trial

**DOI:** 10.1186/s12903-019-0982-3

**Published:** 2020-02-03

**Authors:** Sumio Akifusa, Ayaka Isobe, Kanako Kibata, Akinori Oyama, Hiroko Oyama, Wataru Ariyoshi, Tatsuji Nishihara

**Affiliations:** 1 School of Oral Health Sciences, Faculty of Dentistry, Kyushu Dental University, 2-6-1, Manazuru, Kokurakita-ku, Kitakyushu, Fukuoka, 803-8580 Japan; 2 HA-PPY Co, Ltd., 1041-57, Tsuruhata-cho, Kita-ku, Kumamoto, 861-5513 Japan; 3 Division of Infections and Molecular Biology, Department of Health Promotion, Kyushu Dental University, 2-6-1, Manazuru, Kokurakita-ku, Kitakyushu, Fukuoka, 803-8580 Japan

**Keywords:** Dental plaque, Electric toothbrush, Personal hygiene performance, Quantitative light-induced fluorescence, Randomized controlled trial

## Abstract

**Background:**

To evaluate the efficacy of a newly developed electric toothbrush in reducing dental plaque via a quantitative light-induced fluorescence-digital (QLF-D)-applied visualisation system in the brush head.

**Methods:**

Participants included 20 adults aged 19 to 28 years. Participants were randomly assigned either (i) an electric toothbrush with a monitor to visualise red-fluorescent dental plaque via a camera built into the brush head (monitor usage group, *n* = 10) or (ii) an electric toothbrush without a monitor (monitor-non-use group, *n* = 10). The amount of dental plaque was assessed by personal hygiene performance (PHP) at baseline and 1 week later.

**Results:**

In the monitor-usage group, PHP score was significantly lower at the 1-week follow-up than at baseline (6 vs 16; range, 0–12 vs 13–21; *P* = 0.029). This change was not observed in the monitor-non-use group (14 vs 13; range, 6–21 vs 2–26; *P* = 0.778). After 1 week, the change in PHP scores in the monitor usage group was significantly greater than that in the monitor non-use group (− 10 vs 0; range, − 21 to 9 vs − 8 to 16; *P* = 0.021).

**Conclusions:**

Our results clearly demonstrate that brushing teeth while looking at a monitor that depicts red-autofluorescent dental plaque via application of QLF-D improved the efficacy of dental-plaque removal relative to brushing teeth without a monitor.

**Trial registration:**

Trial registration number: UMIN000033699.

Name of registry: Study on effect of new devise for oral care on dental plaque clearance.

Date of registration: 8th September 2018.

Status of registration: Completed.

## Background

Quantitative light-induced fluorescence-digital (QLF-D)-applied visualisation can be used to detect dental plaque on both human and animal teeth [1–7]. Red auto-fluorescence of dental plaque reflects endogenous porphyrins associated with products of microbiota metabolism [8, 9]; the fluorescence of porphyrins corresponds to characteristic bands of absorbance between wavelengths of 390 nm and 425 nm, named Soret bands [10, 11]. Although not all dental plaque on the teeth showed red fluorescence [12, 13], old and matured dental plaque does when exposed to light of approximately 400-nm wavelength. Previous studies revealed that the amount of red auto-florescent plaque is closely related to incidence of caries [13–15] and gingivitis [16].

Electric toothbrushes have rapidly developed as an established alternative to manual tooth brushing over the past three decades [17–19] and are now widely available; they can be classified into three types: oscillating-rotating, sonic, and ultrasonic. Evidence from multiple studies suggests that oscillating-rotating brushes reduce plaque and gingivitis more than do sonic brushes [20–22]. Compared with manual toothbrushes, electric toothbrushes removed dental plaque more efficiently among visually impaired school students [23], residents of nursing homes [24], and generally healthy people [20]. Most recently, we developed an oscillating-rotating electric toothbrush with a QLF-D built-in brush head that includes a light source and camera. Users of this toothbrush can observe red-fluorescent dental plaque on the tooth surface while brushing their teeth in real-time via a monitor, such as a tablet or smartphone. The purpose of the current study was to evaluate the efficacy of this system in the reduction of dental plaque relative to the use of an electronic toothbrush in the absence of a monitor among generally healthy participants without clinical gingivitis over the course of a one-week period. The present study is, to the best of our knowledge, the first to report on the use of this novel system comprising an oscillating-rotating electric toothbrush with a QLF-D built-in brush head and monitor.

## Methods

### Study participants

The present investigation enrolled 20 dental students, aged 19 to 28 years. All participants were school of dentistry students. All researchers were staff members of the school of oral health sciences. As such, the students did not depend on the researchers for their grades. The experiment was explained to all participants prior to its commencement, and both verbal and written consent to participate were provided. This study was conducted with the approval of the ethics review board of Kyushu Dental University (No.: 18–8).

### Study design

**Fig. 1:**
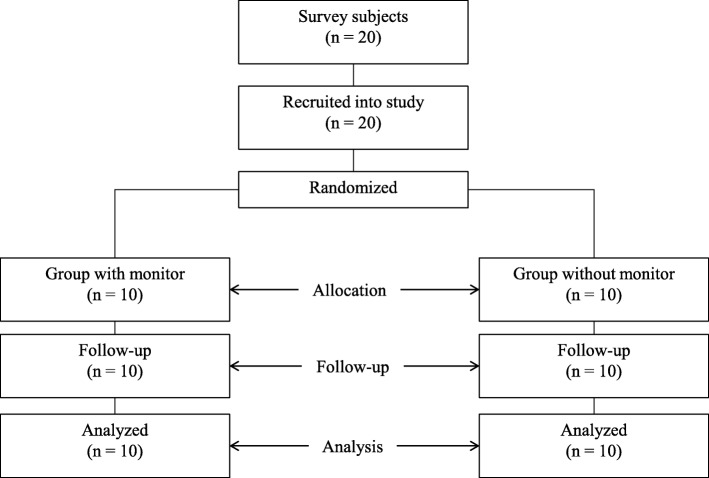
Flow of the study and number of participants at each stage

This was a one-week, single-centre, randomised, two-treatment, examiner-blind, parallel group study (Fig. 1). This randomised controlled trial was performed in accordance with the CONSORT (Consolidated Standards of Reporting Trials) checklist [25]. The trial participants were randomly assigned either 1) an electric toothbrush with a monitor (monitor-usage group; *n* = 10) or 2) an electric toothbrush without a monitor (monitor-non-use group; *n* = 10). Eligible participants were adults aged 18 years or over with healthy gingiva. Exclusion criteria were gingivitis or periodontitis, current antibiotic therapy, smoking and ongoing dental treatment. The study was conducted at Kitakyushu, Japan, in October 2018.

An independent researcher (S. A., who was not the examiner) assigned the participants randomly to either the monitor-usage group or the monitor-non-use group to ensure that the examiner was blinded to both group sampling and assignment. All participants were examined initially and then attended a follow-up examination after one week. Oral examinations were performed from 4 pm to 6 pm. Both groups brushed their teeth with the same dentifrice (Butler Dental Liquid Gel, Sunstar, Osaka, Japan).

The primary outcome was difference in the amount of dental plaque between baseline and follow-up as assessed by personal hygiene performance (PHP, see section 2.4).

### QLR-applied electric toothbrush visualisation system

**Fig. 2:**
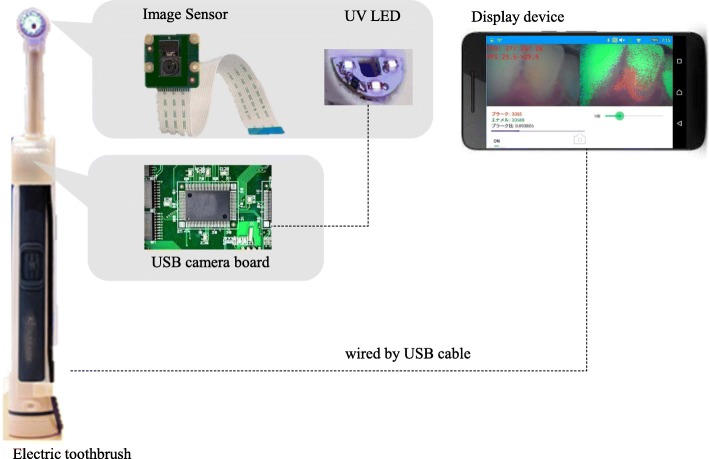
Composition of electric toothbrush with dental plaque visualization system

The newly developed electric toothbrush used in the present experiment is a device incorporating an image sensor with a λ > 520-nm filter and 400-nm wavelength light source with a microcomputer in the head and handle, respectively, in an oscillating-rotating brush (Fig. 2). The electric toothbrush can be connected to a display device, such as a tablet or smartphone, to visualise red-autofluorescent plaque. To classify captured image data as teeth, plaque, or gingiva, each pixel of image data was assigned three elements of colour: hue, saturation, and brightness. The plaque was then visualised as red fluorescence on the display. In addition, users could see the pixel numbers that featured the red-fluorescent plaque.

### Quantitative evaluation of dental plaque

To quantitatively evaluate the dental plaque, the present study used the personal hygiene performance (PHP) [26]. After applying the disclosing agent, surfaces of six teeth (i.e. 16, 11, 26, 36, 31, and 46) were divided into five areas (i.e., three longitudinal thirds, distal, middle, and mesial; the middle third was subdivided horizontally into incisal, middle, and gingival thirds). Two teeth (36 and 46) were examined on their lingual sides, while the other teeth (16, 11, 26, and 31) were examined on their facial sides. The score assigned to each tooth ranged from 0 to 5. Individual scores were obtained by totalling six teeth, and so ranged from 0 to 30.

### Assessment of gingival status

Evaluation of the gingival state was performed with the Gingival Index (GI) according to Löe and Silness on the facial, lingual, distal, and mesial surfaces of six teeth (16, 12, 24, and 36, 32, 44) [27]. The GI of each surface was scored from 0 to 3 according to the severity of the gingival state: 0 indicated normal gingiva; 1, mild inflammation, a slight change in colour, mild alteration of gingival surface structure, and no bleeding on probing (BOP); 2, moderate inflammation, redness, oedema and swelling, and BOP; and 3, severe inflammation, marked redness and oedema, ulceration, and a tendency towards spontaneous bleeding. The mean of the values from all the examined surfaces was calculated. The score of personal GI thus ranged from 0 to 3.

### Questionnaire for self-efficacy of oral health

Self-efficacy of the oral health of participants was assessed using a self-efficacy scale for oral health behaviour (SEOH) [28]. The SEOH questionnaire consisted of 25 items, which were assessed using a five-point Likert scale that addressed four domains: ‘self-efficacy for brushing behaviour’, ‘self-efficacy for daily life habits’, ‘self-efficacy for psychological control’, and ‘self-efficacy for dental check-up’. The score of SEOH ranged from 0 to 125, where a lower score indicated a more positive self-efficacy.

### Study procedures

Initially, the PHP, GI, and SEOH of all participants were assessed. Participants in the two study groups were instructed on the use of the electric toothbrush, and the monitor-usage group received an additional explanation concerning the operation of the monitor. To ensure blinding with regard to monitor usage, the monitor-non-use group first performed the procedures for 1 week, followed by the monitor-usage group. Participants were informed that the toothbrush was newly developed and that the aim of this study was to evaluate the plaque-removable effect of this toothbrush. Neither group was aware of the toothbrush used in the other group. Each group was assessed at the end of the one-week trial. Participants used the toothbrush at home.

### Statistical analysis

All values are presented as median with upper and lower limits. The internal consistency of SEOH was assessed with Cronbach’s α coefficients. The Mann-Whitney *U* test was used to compare variables or extent of change in PHP between groups. The Kruskal-Wallis test was used to compare both groups before and after examination. The statistical analyses were performed using SPSS (version 22; IBM Inc., Armonk, NY, USA). Two-tailed *p*-values were calculated in all analyses. The alpha level for significance was set at 0.05.

## Results

All participants were non-smokers. Cronbach’s α was 0.825 for SEOH. No adverse events were observed or reported during the study. Differences in sex ratio (monitor use group: man 6, woman 4; monitor non-use group: man: 4, woman 6), age (monitor use group: 21; monitor non-use group: 21) were nonsignificant. GI, PHP, and SEOH scores between the two groups were also nonsignificant. Comparisons of each variable at baseline and follow-up are presented in Table 1. In the monitor-usage group, PHP scores had significantly decreased by the follow-up exam (6 to 16; range, 0–2 and 13–21, respectively; *P* = 0.029). While in the monitor non-use group, PHP scores did not change significantly between baseline and follow-up (14 to 13; range, 6–20 and 2–26, respectively). There were no significant differences in the GI and SEOH scores between baseline and follow-up regardless of monitor usage.

**Table 1:** Comparison of each variable at baseline and at follow-up

variables	monitor usage	Baseline/ Follow-up	median (min-max)	*p*^a^	*p*^b^
GI	Yes	Baseline	0.17 (0–0.67)	0.715	
		Follow-up	0.08 (0–0.50)		
	No	Baseline	0 (0–0.59)	0.786	0.370
		Follow-up	0.17 (0–0.50)		0.673
PHP	Yes	Baseline	16 (13–21)	0.029	
		Follow-up	6 (0–12)		
	No	Baseline	14 (6–20)	0.778	0.167
		Follow-up	13 (2–26)		0.114
SEOH	Yes	Baseline	33 (21–49)	0.574	
		Follow-up	42 (22–56)		
	No	Baseline	38 (22–50)	0.310	0.114
		Follow-up	43 (31–51)		0.277

^a^Wilcoxon signed rank test to compare between baseline and follow-up in monitor usage group or monitor non-usage group, respectively

^b^Mann-Whitney test to compare between yes and no regarding monitor usage in baseline or follow-up, respectively

## Discussion

The present study evaluated the effects of the newly developed electric toothbrush with a QLF-D-applied dental plaque visualisation system on the removal of dental plaque. As this device can aid in visualising dental plaque on tooth surfaces in real time, users of this device can easily observe dental plaque remaining on a tooth surface as they brush via a monitor, for example, on a tablet or smartphone. Our results clearly demonstrate that brushing teeth while looking at a monitor that depicts red-autofluorescent dental plaque improves the efficacy of dental-plaque removal relative to brushing teeth without a monitor.

The usage of the new device did not influence gingivitis status as assessed via GI; nevertheless, this finding suggests that the new device had no harmful impact on the gingiva. Participants in this study were dental students and did not have clinical gingivitis. To evaluate the effect of this device on gingivitis, a trial including patients with clinical gingivitis is required.

Self-efficacy for oral health as assessed by SEOH did not change from baseline to follow-up. A previous study on adolescents attending school demonstrated that an 8-week oral health education program using Qscan, a device based on QLF-D, significantly reduced plaque index and improved oral health knowledge, attitude, and behaviour [29]. Participants in the present study were not provided with any educational information on oral health behaviour and tooth brushing was done individually. Although a period of 1 week might have been too short to modify behaviour or self-efficacy of oral health, our research in context with the aforementioned prior findings suggests that the use of the visualisation system alone might be insufficient to improve oral health-related attitude, behaviours, or self-efficacy.

Due to the camera device, the height of head of the toothbrush was higher than that of a common electric toothbrush. However, the unique shape did not have a harmful effect, as a result of GI.

## Limitations

This study has several limitations. First, the trial period was short. Although the major outcome of the present study was the effect of tooth brushing while visualising red-auto-fluorescent dental plaque on reducing dental plaque, the study’s time course was too brief to observe any effect on gingiva or self-efficacy of oral health. Further research on the therapeutic effects of the new device on gingivitis conducted over a longer period is needed. Second, all participants were young dental students. They had good eyesight and no impediments to necessary arm movements. To confirm the applicability of our findings to other age groups, future experiments should enrol middle- and old-aged participants. In addition, the participants featured good oral health-related self-efficacy for oral hygiene at baseline. Assessments of the monitor system should be performed with participants unrelated to dentistry to observe its effect on oral-health-related behaviour and self-efficacy. Third, to avoid obscuring the visual field on the tooth surface while brushing, a dentifrice without a foaming agent was supplied to the participants. As such liquid-type dentifrices account for only 22% of dentifrice-consumption in Japan, further studies on more commonly used types of dentifrice are required to validate our findings.

## Conclusion

Despite the limitations of the present study, tooth brushing while looking at a monitor that depicts red-fluorescent dental plaque with application of a QLF-D system efficiently reduced dental plaque relative to brushing teeth without a monitor.

## Data Availability

The datasets used and/or analysed during the current study are available from the corresponding author on reasonable request.

## Abbreviations

GI: Gingival index
PHP: Personal hygiene performance
QLF-D: Quantitative light-induced fluorescence-digital
SEOH: Self-efficacy scale for oral health behaviour
